# Association of 45-bp ins/del polymorphism of uncoupling protein 2 (UCP2) and susceptibility to nonalcoholic fatty liver and type 2 diabetes mellitus in North-west of Iran

**DOI:** 10.1186/s13104-021-05586-9

**Published:** 2021-05-06

**Authors:** Saleheh rezapour, Shiva Ahdi khosroshahi, Hadi Farajnia, Fatemeh Mohseni, Manouchehr Khoshbaten, Safar Farajnia

**Affiliations:** 1 Biotechnology Research Center, Tabriz University of Medical Sciences, Tabriz, Iran; 2 Drug Applied Research Center, Tabriz University of Medical Sciences, Tabriz, Iran; 3 Immunology Research Center, Tabriz University of Medical Sciences, Tabriz, Iran; 4 Department of Medical Genetics, Faculty of Medicine, Tabriz University of Medical Sciences, Tabriz, Iran; 5 Nutrition Research Center, Tabriz University of Medical Sciences, Tabriz, Iran

**Keywords:** UCP2, 45 bp I/D polymorphism, NAFLD, T2DM

## Abstract

**Objective:**

Uncoupling protein 2 (UCP2) plays a crucial role in energy homeostasis via insulin secretion regulation, free fatty acid concentrations, and lipid metabolism. This study aimed to investigate the association of 45-bp ins/del polymorphism of UCP2 with susceptibility to NAFLD (Non-Alcoholic Fatty Liver Disease) and T2DM (Type 2 Diabetes Mellitus). DNA was extracted from the white blood cells of the subjects, and the gene polymorphism was determined using polymerase chain reaction (PCR). In this study, 72 patients with NAFLD, 71 healthy individuals as control, 80 patients with T2DM, and 77 healthy controls were enrolled in the study.

**Results:**

A higher prevalence of insertion/insertion genotype was observed in T2DM patients compared to the controls (p- value˂ 0.05). There was no difference in genotype distribution between NAFLD patients and controls (p-value > 0.05). NAFLD patients with D/D, D/I genotype had higher triglyceride, ALT, and AST levels; however, their HDL levels were lower than healthy controls. Patients with T2DM with D/D or D/I genotype also had significantly higher fasting serum glucose (FSG). While we found an association between the 45 bp I/D polymorphism in 3ʹUTR of UCP2 and T2DM, no correlation between this polymorphism and NAFLD was identified.

## Introduction

A wide range of studies has been suggested the existence of strong associations among nonalcoholic fatty liver disease (NAFLD), obesity, type 2 diabetes mellitus (T2DM), dyslipidemia, hypertension, cardiovascular disease, and sometimes even hepatocellular carcinoma [1–3]. NAFLD is considered a significant problem that affects approximately 10–30% of the general population of different ethnicities across all world regions [4, 5]. Intriguingly, the prevalence of NAFLD is 49–62% in patients with T2DM, and 18–33% of patients with NAFLD have T2DM [6–8]. While several studies have suggested that T2DM is an independent risk factor for NAFLD, T2DM can lead to NAFLD in the presence of TG accumulation in liver tissue [9, 10].

Uncoupling protein 2 (UCP2) is a mitochondrial inner-membrane anion carrier protein involved in energy homeostasis, regulation of insulin secretion, free fatty acid (FFA) concentrations as well as lipid metabolism [11, 12]. The UCP2 is widely expressed in human tissues, containing white adipose tissue, skeletal muscle, pancreatic islets, and the central nervous system [13, 14]. Due to the unique capabilities of UCP2 to promote lipid accumulation in the liver, stimulation of protective neural mechanisms in acute ethanol intake [15, 16], and reinforcement of insulin resistance [17], its putative role in the pathophysiology of liver disease and obesity has been accepted. Interestingly, research studies have demonstrated a correlation between polymorphisms within the UCP2 gene and metabolic diseases, particularly T2DM and obesity [18]. Based on studies, modifying the expression of genes that regulate UCP-2 expression and functions is a promising therapeutic approach for controlling insulin resistance, obesity, and body-weight gain or body mass index (BMI) [19]. Notably,, genetic polymorphisms in UCP2, particularly 45 bp deletion/insertion (D/I), have been reported to be associated with obesity, BMI, and T2DM in the general population [20].

Because of their high prevalence, increased morbidity and mortality, and social and economic burden, NAFLD and T2DM constitute a significant public health problem [21–23]. Accordingly, recognizing the molecular base of the NAFLD and T2DM is required to open a new landscape toward novel and practical therapeutic approaches. To our knowledge, there are no data on the relationship between the 45-bp D/I polymorphism in the UCP2 gene, NAFLD, and T2DM in the population of North-West of Iran. The purpose of the current study was to investigate the associations between 45-bp ins/del polymorphism and susceptibility to NAFLD and T2DM in a North-West of Iran population or not.

## Main text

### Methods

#### Patient recruitment

In this case–control study, 72 patients with NAFLD (age range: 20–50 years), 71 healthy controls, 80 patients with T2DM, and 77 healthy individuals as control were enrolled. Controls were matched on age and ethnicity. The patients whose differential diagnosis was confirmed by a physician according to ultrasonography and biochemical tests were referred to the outpatient clinics of Tabriz University of Medical Sciences, Tabriz, Iran. NAFLD patients’ inclusion criteria were Iranian ancestry and unrelated, age range between 20 and 50 years old, having body mass index (BMI) between 25 and 39 kg/m^2^, and lack of alcohol consumption. The exclusion criteria for controls were using medications, including metformin, corticosteroids, amiodarone, and/or valproate in the past three months, any history of acute and chronic liver diseases, viral hepatitis, hemochromatosis, Wilson disease, any autoimmune or endocrine disorders, and participation in the weight loss diets for at least three months before joining the study. Type 2 diabetic patients were identified by an endocrinologist based on biochemical tests. The T2DM patients and control group were selected from individuals in the age range of 30 and 70. They hadn’t type 1 diabetes and a history of insulin injection. The Control group also had no history of diabetic disease. Subjects provided written informed consent after a full explanation of the research outline. The study protocol was reviewed and approved by the Ethics Committee of the Tabriz University of Medical Sciences.

#### Biochemical measurements

Fasting serum glucose (FSG), total cholesterol (TC), high-density lipoprotein cholesterol (HDLC), triglycerides (TG), aspartate aminotransferase (AST), and alanine aminotransferase (ALT) concentrations were checked using kits on the Abbott ALCYON 300 auto-analyzer (Abbott Laboratories, Inc) after fasting for more than 10 h. All of the used biochemical parameters are listed in Table 1.

**Table 1:** Demographic and biochemical characteristic of NAFLD and Diabetic type 2 study groups

Variable	NAFLD group	Control group	Mean difference (95% CI)	P value
Sex, No. (%)				0.402
Female	37 (51.4%)	42 (59.2%)		NA
Male	35 (48.6%)	29 (40.8%)		NA
Age	42.00 (35.5 to 49)	40 (33 to 45)	NA	0.167
BMI ( kg/m^2^)	31.69 (4.17)	31.37 (3.96)	3.164 (− 1.03 to 1.67)	0.644
BMR(kcal/d)	1732 (1502 to 2017)	1600 (1426 to 1994)	NA	0.329
FAT (%)	33.6 (24.9 to 39.6)	35.6 (27.35 to 39.75)	NA	0.297
FFM (%)	56.9 (49.35 to 68.2)	52.7 (46.25 to 68)	NA	0.298
FSG (mg/dl)	91.21 (11.35)	89.77 (9.91)	1.352 (− 2.15 to 4.85)	0.424
Cholesterol (mg/dl)	183.28 (36.64)	188.17(30.72)	− 4.741 (− 15.92 to 6.44)	0.391
TG (mg/dl)	152 (114 to 225)	130 (84 to 207)	NA	0.039
HDL (mg/dl)	45 (33.5 to 51)	48 (38.5 to 58)	NA	0.036
LDL (mg/dl)	104.16 (35.98)	111.74 (28.27)	− 7.5706 (− 18.33 to 3.18)	0.166
ALT (IU/L)	44 (26.5 to 66.5)	25 (19 to 33.5)	NA	0.00
AST (IU/L)	32 (20.5 to 38.5)	23 (19 to 28)	NA	0.00

Variable	Diabetic group	Control group	Mean difference (95% CI)	P value
Sex, No. (%)				0.351
Female	36 (45%)	29 (37.7%)		NA
Male	44 (55%)	48 (62.3%)		NA
Age	55.5 (7.02)	53.21 (8.51)	2.29 (− 0.16 to 4.75)	0.06
BMI ( kg/m^2^)	24.26 (3.78)	25.38 (3.23)	− 1.116 (− 2.22 to − 0.004)	0.05
FSG (mg/dl)	146 (127.5 to 206.5)	90 (84.0 to 97.0)	NA	0.00
CHOL (mg/dl)	189.32 (50.16)	188.91 (36.49)	0.414 (− 13.99 to 14.82)	0.95
TG (mg/dl)	183.0 (122.5 to 255.0)	130.0 (88.5 to 212.0)	NA	0.002
LDL (mg/dl)	95.6 (23.54)	107.54 (29.71)	− 12.385 (− 24.177 to 0.59)	0.04
HDL (mg/dl)	40.0 (35.0 to 45)	48.0 (39.0 to 58.0)	NA	0.001

NAFLD group: CI, confidence interval; NA, not applicable. The P-value for sex is based on chi squares; for BMI, FSG, cholesterol, and LDL are based on independent t-testing; otherwise, based on Mann–Whitney testing. BMI, FSG, cholesterol, LDL values are presented based on mean (SD) values; data for other variables are presented based on median (P25–P75). Diabetic group: P-value for sex is based on chi-squares and for FSG, TG, HDL values are based on Mann–Whitney testing; other parameters are based on independent t-testing using an equal variable. FSG, TG, HDL values are presented based on median (P25–P75); data for other variables are presented based on mean (SD) values

#### DNA isolation and polymerase chain reaction (PCR)

Genomic DNA was extracted from whole blood by using the salting-out method [24]. We selected the SNP in the UCP2 gene from published literature and the Database of Single Nucleotide Polymorphism (dbSNP) at the NCBI website (http://www.ncbi.nlm.nih.gov/SNP). The SNP genotyping was performed by PCR. DNA fragments related to 45-bp ins/del polymorphism were amplified by primers: 5ʹ- TTCTCCGCTTGGGTTCCTG -3ʹ as the forward primer and 5ʹ-CACTGTCAAATGTCAACTCCACC-3ʹ as the reverse primer. The PCR primers were designed by Gene Runner software (version 3.01), based on GenBank coding sequence NG_011478, from the National Center for Biotechnology Information (NCBI).

#### Statistical analysis

All statistical analyses were conducted using the SPSS statistical package ver. 22.0 (SPSS Software, Chicago, IL, USA). The Distributions of categorical variables in groups compared using the chi-squared test. Kolmogorov–Smirnov and Shapiro–Wilk tests were performed to determine the normal distribution of quantitative variables. We used the t-test for comparing quantitative data between two groups that had a normal distribution and the Mann–Whitney test for data that had abnormal distribution. Odd Ratio calculated by logistic regression for genotypes and alleles that adjusted by gender and age. The evaluation of continuous variables changes between different UCP-2 genotypes was carried out by analyzing covariance (ANCOVA) with modification effects of age and sex.

### Results

#### Anthropometric and laboratory data of the study population

Table 1 presents the clinical and demographic data of patients enrolled the study (NAFLD; n = 72, controls; n = 71) (T2DM; n = 80, controls; n = 77). Subjects with NAFLD comprised 35 (48.6%) men and 37 (51.4%) women, with a mean age of 40. Their mean difference of BMI was 3.164 kg/m, and there were no significant differences in age, gender, BMI, and BMR between the case and control groups. Subjects with T2DM comprised 36 (45%) men and 44(55%) women, with a mean age of 54 years, and their mean difference of BMI was − 1.12 kg/m^2^. Also, there were no significant differences in age or gender among groups. However, we found that patients with NAFLD had significantly higher triglyceride, ALT, AST, and lower HDL than healthy controls (p ≦ 0.05 for all values). Moreover, patients with T2DM had significantly higher FSG, TG, and lower LDL and HDL levels than healthy controls (p ≦ 0.05 for all values).

#### Genotypes and allele frequencies of polymorphism

The frequency of 45 bp D/I genotypes from more to less was belonged to DD homozygote, DI heterozygotes, and II homozygotes, respectively. PCR products for deletion and insertion alleles were 310 bp and 355 bp, respectively. We observed no significant differences in the genotypic distribution or allelic frequency of 45 bp Ins/Del between the NAFLD and control groups (p value > 0.05). However, UCP-2 I/I genotype (p = 0.025) and UCP-2 I allele (p = 0.004) were associated with susceptibility to T2DM. Subgroup analysis revealed that the proportion of subjects with homozygous genotype D/D was higher in control cases (57.7%) than in patients with NAFLD (51.4%) and that the ratios of heterozygous D/I were higher in NAFLD (40.3%) than in control groups (32.4%). However, the distribution of genotypes and allelic frequency was not significantly different between NAFLD patients and control subjects (see Table 2). Subgroup analysis in T2DM subjects also revealed that the frequency of subjects with homozygous genotype D/D was higher in control cases (64.9%) than in patients with T2DM (46.3%). The frequency of subjects with heterozygotes D/I and homozygous I/I were higher in patients with T2DM than in control cases (33.8% vs. 26.0% and 20.0% vs. 9.1%, respectively).

**Table 2:** Association of the 45-bp I/D polymorphism of UCP2 gene in NAFLD and Diabetes mellitus type 2 in the Study Population

45 bp Ins/Del	NAFLD				T2DM			
Genotypes	Case	Control	OR (95% CI)	P Value	Case	Control	OR (95% CI)	P Value
D/D	37 (51.4%)	41 (57.7%)	Ref	–	37 (46.3%)	50 (64.9%)	Ref	–
D/I	29 (40.3%)	23 (32.4%)	1.39 (0.69 to 2.81)	0.35	27 (33.8%)	20 (26.0%)	1.82 (0.89 to 3.73)	0.10
I/I	6 (8.30%)	7 (9.9%)	0.95 (0.29 to 3.08)	0.93	16 (20.0%)	7 (9.1%)	3.088 (1.15 to 8.26)	0.025
Alleles								
D	103 (71.53)	105 (73.94)	Ref	–	101 (63.12%)	120 (77.92%)	Ref	–
I	41 (28.47%)	37 (26.05)	1.13 (0.67 to 1.90)	0.647	59 (36.87%)	34 (22.07%)	2.06 (1.25 to 3.39)	0.004

OR; odds ratio, CI; confidence interval, D: Deletion, D/D: Deletion/Deletion, I: Insertion, I/I: Insertion/Insertion. P value is based on logistic regression analysis

#### Relationship between genotypes and laboratory data

Evaluation of changes in continuous variables among different UCP2-45 bp D/I genotypes showed that HDL level in subjects with D/D genotypes was significantly lower in NAFLD patients than in the control group. Also, ALT and AST levels in those with D/D and D/I genotypes were significantly higher in the NAFLD patient group compared to the control group (Table 3). Moreover, in T2DM subjects with D/D and D/I genotype, the serum level of FSG was significantly higher than healthy control. In individuals with I/I genotype, the concentrations of cholesterol and LDL in serum were significantly lower in T2DM patients than in the healthy group (Table 3). No significant differences were observed between other clinical or laboratory characteristics and genotypes.

**Table 3:** Assessment of study variables based on UCP2 45 bp Ins/Del genotypes in NAFLD and T2DM patients with healthy groups

Variable	Genotypes	NAFLD group	Control group	Mean difference (95% CI)	P value
BMI ( kg/m^2^)	D/D	31.95 (4.28)	31.01 (3.52)	0.69 (− 1.02 to 2.41)	0.424
	D/I	31.39 (4.12)	32.17 (4.79)	− 0.15 (− 2.65 to 2.34)	0.901
	I/I	31.56 (4.33)	31.52 (5.07)	− 0.56 (− 6.34 to 5.20)	0.829
BMR(kcal/d)	D/D	1658.0 (1488.0 to 1955.0)	1600 (1403.5 to 2022.5)	NA	0.52
	D/I	1812.0 (1592.5 to 2023.5)	1670.0 (1434.5 to 1999.0)	NA	0.803
	I/I	1580.00 (1462.5 to 2124.5)	1576.0 (1426.0 to 1830.0)	NA	0.241
FAT (%)	D/D	36.3 (24.7 to 41.6)	32.4 (27.7 to 38.15)	NA	0.83
	D/I	30.70 (25.25 to 36.25)	38.60 (26.65 to 42.95)	NA	0.246
	I/I	38.7 (22.5 to 39.65)	36.00 (26.30 to 39.5)	NA	0.39
FFM (%)	D/D	55.4 (47.7 to 65)	53.2 (45.8 to 68.3)	NA	0.71
	D/I	60.90 (52.35 to 68.30)	53.60 (46.05 to 67.70)	NA	0.983
	I/I	52.20 (48.05 to 72.10)	50.90 (46.20 to 61.90)	NA	0.66
FSG (mg/dl)	D/D	92.34 (11.45)	90.22 (10.26)	1.569 (− 3.35 to 6.49)	0.52
	D/I	90.76 (10.263)	90.29 (9.52)	− 0.717 (− 6.65 to 5.22)	0.809
	I/I	88.40 (16.11)	86.86 (9.20)	2.861 (− 16.03 to 21.7)	0.736
Cholesterol (mg/dl)	D/D	188.14 (41.79)	187.51 (29.750)	− 0.66 (− 17.28 to 15.94)	0.936
	D/I	179.79 (28.74)	191.52 (31.73)	− 10.61 (− 28.93 to 7.699)	0.249
	I/I	165.00 (13.26)	185.00 (38.30)	− 27.17 (− 71.13 to 16.78)	0.192
TG (mg/dl)	D/D	154.00 (114 to 225)	117.00 (77 to 213)	NA	0.066
	D/I	152.00 (100.0 to 234.5)	142.00 (103.0 to 192.50)	NA	0.913
	I/I	144.0 (135.0 to 184.5)	140.0 (69.0 to 214.0)	NA	0.762
HDL (mg/dl)	D/D	45.00 (33.00 to 54.0)	48.00 (38.5 to 58.0)	NA	0.026
	D/I	45.00 (34.0 to 48.0)	45.00 (38.0 to 58.0)	NA	0.505
	I/I	45.0 (40.0 to 56.5)	54.00 (34.0 to 59.0)	NA	0.507
LDL (mg/dl)	D/D	103.120 (39.43)	110.62 (26.53)	− 9.0 (− 24.28 to 6.26)	0.244
	D/I	101.876 (26.44)	113.26 (29.78)	− 8.83 (− 25.64 to 7.98)	0.296
	I/I	86.08 (8.82)	108.25 (35.60)	− 28.82 (− 67.73 to 10.08)	0.126
ALT (IU/L)	D/D	44.00 (30.0 to 67.0)	22.00 (17.5 to 33.00)	NA	0.00
	D/I	50.00 (26.00 to 69.5)	29.00 (23.00 to 34.50)	NA	0.002
	I/I	27.00 (19.50 to 42.5)	31.00 (23.00 to 35.00)	NA	0.866
AST (IU/L)	D/D	32.00 (20.0 to 41.0)	21 (17.5 to 27.5)	NA	0.00
	D/I	33.00 (22.00 to 38.50)	24.00 (21.50 to 27.50)	NA	0.012
	I/I	19.00 (19.00 to 27.00)	27.00 (21.00 to 34.00)	NA	0.095

Variable	Genotypes	Diabetic group	Control group	Mean difference (95% CI)	P value
BMI ( kg/m^2^)	D/D	24.81 (4.32)	25.30 (3.13)	− 0.598 (− 3.19 to 1.99)	0.646
	D/I	24.89 (4.344)	26.007 (2.81)	− 2.511 (− 7.03 to 2.01)	0.263
	I/I	22.95 (2.55)	25.65 (3.45)	− 4.527 (− 10.644 to 1.59)	0.13
FSG (mg/dl)	D/D	133.0 (126.0 to 193.0)	90.0 (84.0 to 96.0)	NA	0.00
	D/I	146.0 (134.0 to 247.0)	94.50 (85.50 to 98.0)	NA	0.006
	I/I	136.0 (117.0 to 190.0)	88.0 (76.0 to 96.0)	NA	0.148
CHOL (mg/dl)	D/D	167.31 (36.48)	184.40 (35.86)	− 23.497 (− 50.37 to 3.37)	0.085
	D/I	206.73 (59.84)	189.25 (28.23)	10.79 (− 40.79 to 62.38)	0.669
	I/I	168.57 (20.065)	185.00 (38.30)	− 56.975 (− 111.68 to − 2.26)	0.043
TG (mg/dl)	D/D	129.0 (107.5 to 179.5)	123.0 (78.0 to 211.0)	NA	0.966
	D/I	278.0 (183.0 to 298.0)	150.0 (108.7^_^ 202.5)	NA	0.444
	I/I	197.0 (115.0 to 215.0)	140.0 (69.0 to 214.0)	NA	0.836
LDL (mg/dl)	D/D	37.46 (5.66)	48.26 (12.37)	− 20.36(− 41.68 to 0.95)	0.061
	D/I	40.64 (7.46)	45.44 (12.49)	− 2.83 (− 40.34 to 34.67)	0.877
	I/I	88.29 (10.193)	108.0 (35.42)	− 54.004 (− 100.93 to − 7.07)	0.028
HDL (mg/dl)	D/D	38.00 (34.50 to 42.5)	49.00 (39.0 to 58.0)	NA	0.263
	D/I	42.0 (34.00 to 47.00)	44.0 (34.5 to 57.5)	NA	0.939
	I/I	40.0 (38.0 to 43.0)	54.0 (34.0 to 59.0)	NA	0.653

D, deletion; I, insertion; CI, confidence interval. P-value is based on ANCOVA with modification effects of age and sex. BMI, FSG, cholesterol, and LDL values are presented based on the mean (SD); other variables are given based on median (P25-P75)

### Discussion

In the current case–control study, The significant result is that UCP2 45 bp D/I polymorphism has the potential to affect liver cell function (as measured by HDL, AST, TG, and ALT levels). We found an association between the 45 bp I/D polymorphism in 3ʹUTR of UCP2 and T2DM; no correlation between this polymorphism and NAFLD was identified. To the best of our knowledge, this is the first report evaluating the prevalence of 45-bp ins/del polymorphism of *UCP2* gene polymorphism and its association with anthropometric and biochemical variables in patients with NAFLD and T2DM.

Ala55Val polymorphism in the exon 4, the -866G/A polymorphism in the promoter region, and the 45 bp D/I polymorphism in the 3’ UTR of exon 8 are three prominently evaluated polymorphisms in the UCP2 gene. Similar to our results, it has been revealed that these variants are associated with different metabolic traits in various populations and ethnic groups [25]. However, some studies have not confirmed the association between these variants and metabolic disorders [26, 27]. The association of 45 bp D/I polymorphism with energy balance was first denoted in Pima Native Americans. While the biological effect of the 3′UTR D/I is not well recognized, its location in the 3′UTR may involve transcript stability or mRNA processing [28].

To our findings, subjects in I/D genotype were 39% and 82% more prone to have NAFLD and T2DM, respectively.

Several studies in different populations have verified that carriers of the I-allele of the UCP-2 gene had a significantly higher BMI and the possibility of obesity [27, 29]. Besides, it was found that individuals with I/D genotype had an increased rate of basal metabolism, high energy expenditure, and lower BMI [30]. Another study revealed that patients with D/D genotype had a remarkable enhancement in total and truncal fat mass and body weight [31]. An investigation carried out on the Korean female population by Yong Hwan Lee et al. revealed that subjects with a 45 bp I allele of UCP2 might have a higher risk of obesity [32]. Other investigations noticed a significant association between this polymorphism and BMI in the European population [33]. Our study found that patients with NAFLD and UCP2 45 bp D/D or D/I genotype had significantly higher TG, ALT, AST, and lower HDL levels than healthy controls. Besides, patients with T2DM and UCP2 45 bp D/D or D/I genotype had meaningfully higher FSG and lower cholesterol and LDL levels than healthy controls, respectively. In Hashemi et al. [34] study there was a correlation between 45-bp I/D polymorphism of UCP2 and metabolic syndrome (MeS) following a case–control study on 151 patients with MeS.

One study on 268 obese and 185 non-obese children and adolescents showed that the I allele may contribute to low HDL cholesterolemia [35]. Various studies have presented UCP-2 45-bp D/I polymorphism association with a higher degree of obesity, insulin resistance, dyslipidemia, and lower adjusted metabolic rate [36, 37]. Conversely, some studies described no correlation between UCP2 45-bp I/D polymorphism and obesity [38, 39]. These inconsistencies are likely due to the small sample size and incomplete coverage of the UCP2 gene variations or potential population-specific influences on metabolic traits [40].

### Conclusion

According to the results, there was an association between UCP2-45 bp I/I polymorphism and elevated risk for T2DM in the population from the North-West of Iran. Moreover, we found that there is no significant association between UCP2-45 bp D/I polymorphism and NAFLD.

## Limitations

These results are specifically valid for the study population, and its generalization to other populations needs further studies.

## Data Availability

All data generated or analysed during this study are included in this published article.

## Abbreviations

NAFLD: Nonalcoholic fatty liver disease
BMI: Body mass index
BMR: Basal metabolic rate
FFM: Fat-free mass
FSG: Fasting serum glucose; HDL, high-density cholesterol
LDL: Low-density cholesterol
ALT: Alanine aminotransferase
AST: Aspartate aminotransferase
TG: Triglyceride

## References

[1] Toshikuni N, Tsuchishima M, Fukumura A, Arisawa T, Tsutsumi M (2017). Associations of fatty liver disease with hypertension, diabetes, and dyslipidemia: comparison between alcoholic and nonalcoholic steatohepatitis. Gastroenterol Res Pract.

[2] Sporea I, Popescu A, Dumitrascu D, Brisc C, Nedelcu L, Trifan A, Gheorghe L, Fierbinteanu BC (2018). Nonalcoholic fatty liver disease: status quo. J Gastrointestin Liver Dis..

[3] Tilg H, Moschen AR, Roden M (2017). NAFLD and diabetes mellitus. Nat Rev Gastroenterol Hepatol.

[4] Dyson JK, Anstee QM, McPherson S (2014). Nonalcoholic fatty liver disease: a practical approach to diagnosis and staging. Frontline Gastroenterol.

[5] Silaghi CA, Silaghi H, Colosi HA, Craciun AE, Farcas A, Cosma DT, Hancu N, Pais R, Georgescu CE (2016). Prevalence and predictors of nonalcoholic fatty liver disease as defined by the fatty liver index in a type 2 diabetes population. Clujul Med.

[6] Jimba S, Nakagami T, Takahashi M, Wakamatsu T, Hirota Y, Iwamoto Y, Wasada TJDM (2005). Prevalence of nonalcoholic fatty liver disease and its association with impaired glucose metabolism in Japanese adults. Diabetic Med.

[7] Fan J-G, Zhu J, Li X-J, Chen L, Li L, Dai F, Li F, Chen S-Y (2005). Prevalence of and risk factors for fatty liver in a general population of Shanghai. China. J Hepatol.

[8] Gupte P, Amarapurkar D, Agal S, Baijal R, Kulshrestha P, Pramanik S, Patel N, Madan A, Amarapurkar AJ (2004). Nonalcoholic steatohepatitis in type 2 diabetes mellitus. J Gastroenterol Hepatol.

[9] Tokita Y, Maejima Y, Shimomura K, Takenoshita S, Ishiyama N, Akuzawa M, Shimomura Y, Nakajima K (2017). Non-alcoholic fatty liver disease is a risk factor for type 2 diabetes in middle-aged Japanese Men and Women. Intern Med.

[10] Bril F, Cusi K (2017). Management of nonalcoholic fatty liver disease in patients with type 2 diabetes: a call to action. Diabetes Care.

[11] Busiello RA, Savarese S, Lombardi A (2015). Mitochondrial uncoupling proteins and energy metabolism. Front Physiol.

[12] Xu H, Hertzel AV, Steen KA, Wang Q, Suttles J, Bernlohr DA (2015). Uncoupling lipid metabolism from inflammation through fatty acid binding protein-dependent expression of UCP2. Mol Cell Biol.

[13] Dalgaard LT, Andersen G, Larsen LH, Sørensen TI, Andersen T, Drivsholm T, Borch-Johnsen K, Fleckner J, Hansen T, Din NJ (2003). Mutational analysis of the UCP2 core promoter and relationships of variants with obesity. Obes Res.

[14] Saleh M, Wheeler M, Chan CBJD (2002). Uncoupling protein-2: evidence for its function as a metabolic regulator. Diabetologia.

[15] Horvath B, Spies C, Horvath G, Kox WJ, Miyamoto S, Barry S, Warden CH, Bechmann I, Diano S, Heemskerk JJB (2002). Uncoupling protein 2 (UCP2) lowers alcohol sensitivity and pain threshold. Biochem Pharmacol.

[16] Jin X, Yu MS, Huang Y, Xiang Z, Chen YP (2017). MiR-30e-UCP2 pathway regulates alcoholic hepatitis progress by influencing ATP and hydrogen peroxide expression. Oncotarget.

[17] Hazlehurst JM, Woods C, Marjot T, Cobbold JF, Tomlinson JW (2016). Nonalcoholic fatty liver disease and diabetes. Metab Clin Exp.

[18] Yang L, Dong Z, Zhou J, Ma Y, Pu W, Zhao D, He H, Ji H, Yang Y, Wang X (2016). Common UCP2 variants contribute to serum urate concentrations and the risk of hyperuricemia. Sci Rep.

[19] Pan H-C, Lee C-C, Chou K-M, Lu S-C, Sun C-Y (2017). Serum levels of uncoupling proteins in patients with differential insulin resistance: a community-based cohort study. Medicine.

[20] Marti A, Corbalan MS, Forga L, Martinez-Gonzalez MA, Martinez JA (2004). Higher obesity risk associated with the exon-8 insertion of the UCP2 gene in a Spanish case-control study. Nutrition.

[21] Chen L, Magliano DJ, Zimmet PZJN (2012). The worldwide epidemiology of type 2 diabetes mellitus—present and future perspectives. Nat Rev Endocrinol.

[22] Koehler EM, Schouten JN, Hansen BE, Hofman A, Stricker BH, Janssen HL (2013). External validation of the fatty liver index for identifying nonalcoholic fatty liver disease in a population-based study. Clin Gastroenterol Hepatol.

[23] Trépo E, Valenti L (2020). Update on NAFLD genetics: from new variants to the clinic. J Hepatol.

[24] Mohammadi S, Farajnia S, Shadmand M, Mohseni F, Baghban R (2020). Association of rs780094 polymorphism of glucokinase regulatory protein with nonalcoholic fatty liver disease. BMC Res Notes.

[25] Liu L, Zhao X, Kang S, Zhang DJG (2013). An association between—866G/A polymorphism in the promoter of UCP2 and obesity: a meta-analysis. Gene.

[26] Qian L, Xu K, Xu X, Gu R, Liu X, Shan S, Yang TJ (2013). UCP2–866G/A, Ala55Val and UCP3–55C/T polymorphisms in association with obesity susceptibility—a meta-analysis study. PLoS ONE.

[27] de Souza BM, Brondani LA, Boucas AP, Sortica DA, Kramer CK, Canani LH, Leitao CB, Crispim DJ (2013). Associations between UCP1–3826A/G, UCP2–866G/A, Ala55Val and Ins/Del, and UCP3–55C/T polymorphisms and susceptibility to type 2 diabetes mellitus: case-control study and meta-analysis. PLoS ONE.

[28] Yu G, Wang J, Xu K, Dong J (2016). Dynamic regulation of uncoupling protein 2 expression by microRNA-214 in hepatocellular carcinoma. Biosci Rep.

[29] Brondani LA, de Souza BM, Assmann TS, Boucas AP, Bauer AC, Canani LH, Crispim D (2014). Association of the UCP polymorphisms with susceptibility to obesity: case-control study and meta-analysis. Mol Biol Rep.

[30] Mutombo PB, Yamasaki M, Shiwaku K (2013). UCP2 I/D modulated change in BMI during a lifestyle modification intervention study in Japanese subjects. Genet Test Mol Biomarkers.

[31] Wang X, Axelsson J, Nordfors L, Qureshi AR, Avesani C, Barany P, Schalling M, Heimbürger O, Lindholm B, Stenvinkel PJ (2006). Changes in fat mass after initiation of maintenance dialysis is influenced by the uncoupling protein 2 exon 8 insertion/deletion polymorphism. Nephrol Dial Transplant.

[32] Lee YH, Kim W, Yu BC, Park BL, Kim LH, Shin HD (2008). Association of the ins/del polymorphisms of uncoupling protein 2 (UCP2) with BMI in a Korean population. Biochem Biophys Res Commun.

[33] Brondani LA, Assmann TS, de Souza BM, Boucas AP, Canani LH, Crispim D (2014). Meta-analysis reveals the association of common variants in the uncoupling protein (UCP) 1–3 genes with body mass index variability. PLoS ONE.

[34] Hashemi M, Rezaei H, Kaykhaei MA, Taheri M (2014). A 45-bp insertion/deletion polymorphism of UCP2 gene is associated with metabolic syndrome. J Diabetes Metab Disord.

[35] Gul A, Ates O, Ozer S, Kasap T, Ensari E, Demir O, Sonmezgoz E (2017). Role of the polymorphisms of uncoupling protein genes in childhood obesity and their association with obesity-related disturbances. Genet Test Mol Biomarkers.

[36] Sreedhar A, Zhao Y (2017). Uncoupling protein 2 and metabolic diseases. Mitochondrion.

[37] Bouillaud F, Alves-Guerra M-C, Ricquier D (2016). UCPs, at the interface between bioenergetics and metabolism. Biochem Biophys Acta.

[38] Papazoglou D, Papathanasiou P, Papanas N, Papatheodorou K, Chatziangeli E, Nikitidis I, Kotsiou S, Maltezos E (2012). Uncoupling protein-2 45-base pair insertion/deletion polymorphism: is there an association with severe obesity and weight loss in morbidly obese subjects?. Metab Syndr Relat Disord.

[39] Zhang M, Wang M, Zhao Z-T (2014). Uncoupling protein 2 gene polymorphisms in association with overweight and obesity susceptibility: a meta-analysis. Meta Gene.

[40] Huang T, Shu Y, Cai Y-D (2015). Genetic differences among ethnic groups. BMC Genomics.

